# Monte Carlo simulation of a multi-leaf collimator design for telecobalt machine using BEAMnrc code

**DOI:** 10.4103/0971-6203.58780

**Published:** 2010

**Authors:** Komanduri M. Ayyangar, M. Dinesh Kumar, Pradush Narayan, Fenedit Jesuraj, M. R. Raju

**Affiliations:** International Cancer Center, Mahatma Gandhi Memorial Medical Trust Hospital, Bhimavaram, AP-534 204, India; 1 Yashoda Hospital, Secunderabad, India

**Keywords:** BEAMnrc, Cobalt-60, multi-leaf collimator, Monte Carlo

## Abstract

This investigation aims to design a practical multi-leaf collimator (MLC) system for the cobalt teletherapy machine and check its radiation properties using the Monte Carlo (MC) method. The cobalt machine was modeled using the BEAMnrc Omega-Beam MC system, which could be freely downloaded from the website of the National Research Council (NRC), Canada. Comparison with standard depth dose data tables and the theoretically modeled beam showed good agreement within 2%. An MLC design with low melting point alloy (LMPA) was tested for leakage properties of leaves. The LMPA leaves with a width of 7 mm and height of 6 cm, with tongue and groove of size 2 mm wide by 4 cm height, produced only 4% extra leakage compared to 10 cm height tungsten leaves. With finite ^60^Co source size, the interleaf leakage was insignificant. This analysis helped to design a prototype MLC as an accessory mount on a cobalt machine. The complete details of the simulation process and analysis of results are discussed.

## Introduction

Clinical radiotherapy using ^60^Co machines are a cost-effective alternative to low energy linear accelerators.[1] Addition of MLC in these machines will make treatment more versatile for implementing 3D CRT plans. There are nearly 300 cobalt machines in India and many more in the world. Most of these machines are similar in design to Theratron-80 models (original design by Atomic Energy Canada Limited (AECL)).

Monte Carlo (MC) simulation can be used to test beams from radiotherapy machines of various designs in terms of their adequacy of shielding and beam transport. For a diverging type collimator Han *et al*,[2] simulated a cobalt beam from Theratron-780C model teletherapy machine using MC computations with Stanford Electron Gamma Shower (EGS) code. They used an original fluence of 2 million photons generated with 2 cm diameter teletherapy source, with depleted uranium collimator. The MC method allows for realistic and accurate results.[3] Instead of actually doing the experiment with expensive equipment, a virtual machine can be simulated and virtual experiments can be performed using MC methods provided the problem is defined accurately and accurate data are used.

MC is vital for development in radiation oncology physics. MC calculations have been used to generate basic data for external beam treatment planning. Finite size pencil beam point spread functions derived from MC data were used in convolution algorithms and analytical anisotropic algorithms. In brachytherapy, basic TG43 data were generated by MC and verified by experiment. In this work, we applied MC methodology to validate the efficacy of a prototype design of MLC for a telecobalt machine.

The cobalt-60 teletherapy machine has been modeled in the past by various investigators.[4–10] Complete cobalt source was simulated by Mora *et al*,[4] using BEAM code as early as in 1999. Earlier, in 1988, Rogers[5] used EGS to calculate the cobalt source in a limited study. The source size used by Rogers was 2 cm diameter. Subsequently, smaller cobalt sources with higher specific activity were available. In 2004, Al-Basheer[8] has conducted a detailed study of the properties of Theratron telecobalt machine using MCNP code. However, he used a source size of 1.5 cm diameter and 3 cm height. More recently there were other studies[7–9] of simulating Theratron sources using MC modeling.

In this paper, we present the building and testing of cobalt-60 teletherapy machine for virtual simulation purposes, calculating depth dose and cross profiles, and generating the leakage data for a typical MLC design.

## Materials and Methods

The current simulation was performed for a Phoenix model cobalt machine manufactured by Kirloskar Technologies, Harihar, India. This is an isocentric machine with source to axis (SAD) distance of 80 cm and source diaphragm distance (SDD) of 45 cm. In addition, a block tray holder with SDD of 55 cm is available. This machine is of similar design to a Theratron −80 ^60^Co machine (M/s Theratronics, Canada).

### EGS and OMEGA BEAM Software

The MC code used in this paper is known as the OMEGA BEAM (Ottawa-Madison Electron Gamma Algorithm) which was originally developed collaboratively by NRC Canada and University of Wisconsin through a grant awarded by NIH in 1994.[3] The BEAM code was specifically developed for radiation therapy beams. It simulates - targets, flattening filters, scattering foils, mirrors, jaws, applicators, etc. The original BEAM code has undergone several revisions and is now called BEAMnrc. The version used in this paper was revised as of February 2007. The BEAM code requires the EGS software as prerequisite, is now called EGSnrcMP, where MP signifies multi-platform. The EGSnrcMP is an extended and improved version of the earlier EGS4 package and incorporates many improvements in the implementation of the condensed history technique for the simulation of charged particle transport and latest low energy cross sections. (See EGSnrcMP link below).

Free versions of the software are available from http://www.irs.inms.nrc.ca/EGSnrc/EGSnrc.html (EGSnrcMP) and http://www.irs.inms.nrc.ca/BEAM/beamhome.html none (BEAMnrc).

Documentation was available in PDF format and was a part of the installation package. Installation packages contained the following modules:

1. EGSnrcMP - the software that tracks the electron-gamma transport
2. BEAMnrc - builds the treatment machines
3. BEAMdp - analyses phase space files created by BEAMnrc
4. DOSXYZnrc - a transport code to process phase space files and compute dose distributions in CT matrix or phantoms.

### ^60^Co Source Configuration and Beam Transport

**Figure 1:**
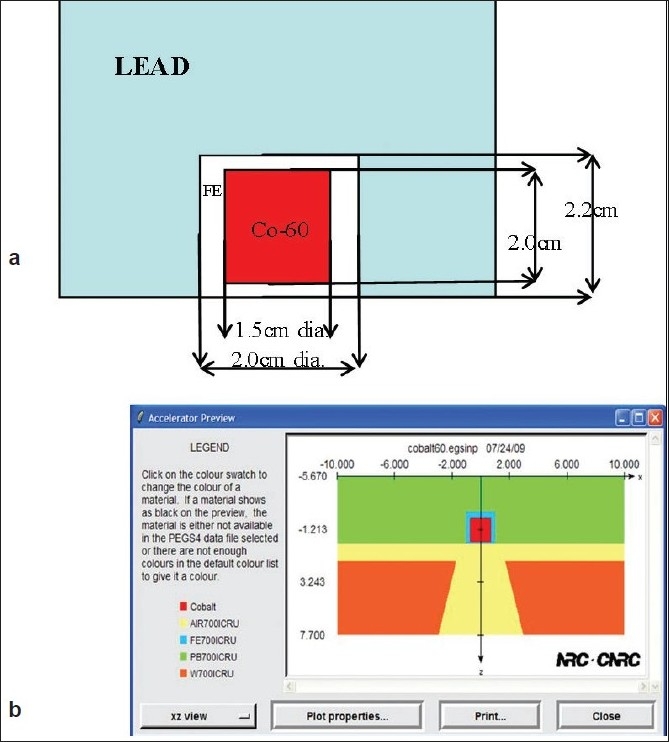
a. Cobalt-60 source configuration modeled in the current MC Study. 1b. Cobalt source housing and primary collimator modeled in step I of MC simulation. Interaction histories at the bottom of the primary collimator were stored in a phase space file

The source size used in the current simulation was 1.5 cm diameter and 2 cm height. The active source was surrounded by steel cladding and a lead shield except for an opening for the beam to exit. Figure 1a shows a diagram of the source housing used for simulation in this work.

All the simulations were carried out on a Dell PC with 2 GHz speed and 512 MB RAM. The current simulation was a three-step process. In the first step, simulation was carried out only for the source, source housing and primary collimator. Results of the simulation were stored as a phase space file at the end of the primary collimator. The primary collimator opening was enough to accommodate the largest field size at the isocenter. The phase space file was scored over a diameter of 10 cm and a total number of 1.7 billion histories were collected in about 160 hours of computational time. This phase space file typically was of size 1.0 Gb. Figure 1b shows the cobalt source housing and primary collimator modeled in step I of the MC simulation. A detailed description of the input file used for this step is given in appendix.

**Figure 2:**
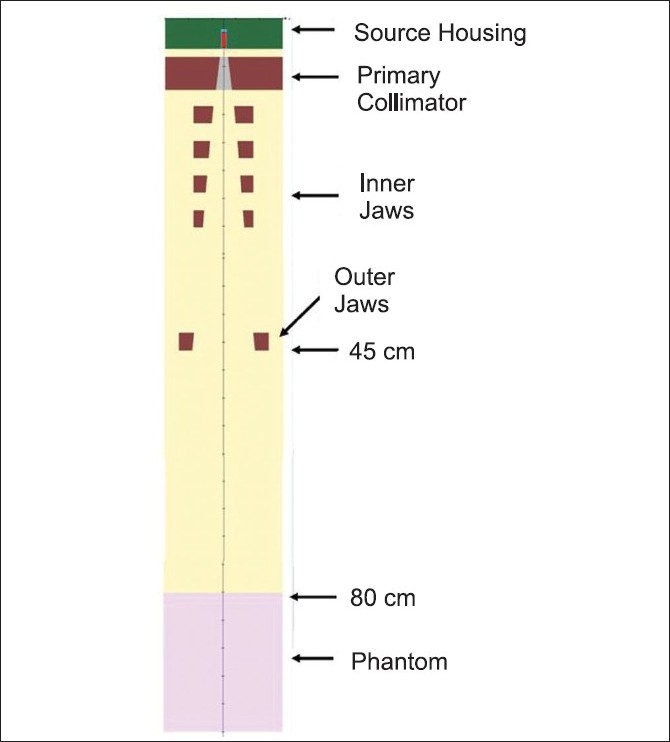
Complete simulation geometry from source to phantom. In step II, the simulation was performed from the bottom of the primary collimator to the water phantom placed at SSD of 80 cm

In step II, simulation was performed using the phase space file generated in step I through the inner and outer jaws and to the top of water phantom placed at 80 cm from source plane. Step II had to be repeated for each new field size. Typically, for a 10 cm × 10 cm field simulation, five billion histories are processed and computational time is around 40 hours. This computation resulted in generation of a phase space file on the top of the water phantom. This file typically was of size 1.2 Gb. In addition, during this process, BEAMnrc calculated the central axis depth dose data in the phantom. Figure 2 shows the complete simulation geometry used in step II with true distances and dimensions of the inner collimator on the Phoenix cobalt machine.

The transport code automatically calculated the percent standard deviation of the mean dose at each scored zone. Selection of the number of histories was based on reducing this value to less than 1%. In step III the phase space file from the previous step was given as input to DOSXYZnrc program which continues the transport of phase space particles into the phantom defined as 3D voxels of size 2.5 × 2.5 × 5.0 mm^3^. The 5 mm grid size was in the direction of depth. Typically this process computed 4.7 billion histories in 116 hours. From the 3D dose matrix generated by DOSXYZnrc, depth dose and beam profile data were derived. In addition, cross sectional data was extracted to generate isodose distribution in planes perpendicular to the beam.

### MLC Fabrication:

Our current design of the MLC for our cobalt machine consisted of 20 leaf pairs of 15 cm leaf length to cover an area of 20 cm × 20 cm field size with a 5 cm leaf over travel from the central axis. The leaf banks would be mounted on 1 cm thick Lucite plate, which was at a distance of 55 cm from the source, attached to the tray holder with two aluminum brackets. The total weight of the collimator with leaves made of low melting point alloy (LMPA), also known as Cerrobend, would be about 30 kg. The design of the proposed MLC is general enough such that when the collimator is built, it can be fitted on to similar machines.

**Figure 3:**
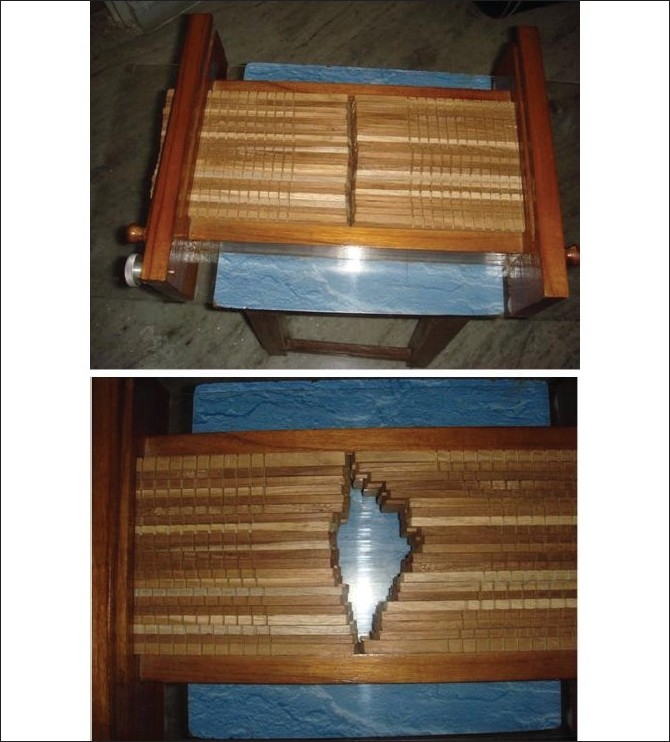
Cobalt MLC prototype design fabricated as wooden model a) There are 20 leaves that project 1cm width at isocenter. b) Leaves are adjusted to depict a treatment port.

We first constructed a wooden model with the help of local carpenters. The model allowed engineers to grasp the functionality and foresee engineering issues. Figure 3 shows a picture of this wooden model. In the current study, prototype leaves were made with both LMPA and lead metal.

**Figure 4:**
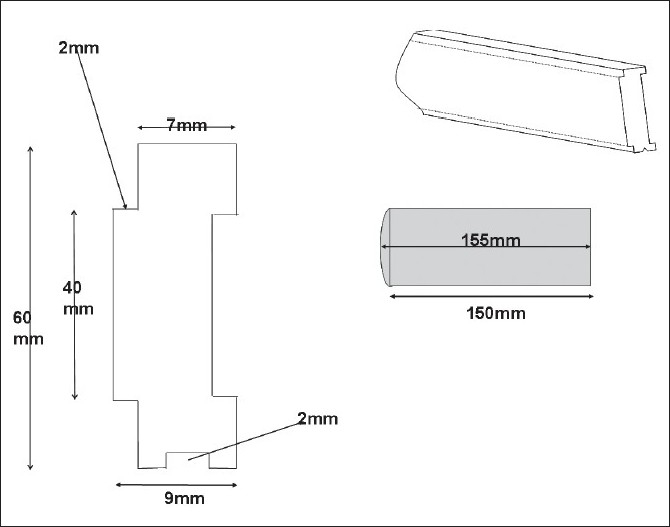
Cross section of the MLC leaf showing height, tongue and groove dimensions. Also drawn is the length and rounded edge of the leaf

In the current design, the leaf height was arbitrarily chosen as 6 cm based on 2% leakage for lead. The leaf thickness (width) was 7 mm projecting a size of 1 cm at the isocenter. The leaf had a rounded edge with a 9.25 cm radius of curvature. The tongue and grooves were 2 mm wide and 4 cm height. Figure 4 shows the cross section and other dimensions of the leaf. The leaf had an additional groove in the bottom to help make the leaf travel on a rail. In addition, the tongue, the groove and the aluminum bracket helps to hold it in position.

This paper addresses the radiation characteristics of this design using MC method. For simulation, the MLC leaf was at a distance of 48 cm from the source. To compare the current MLC design with a near perfect MLC at the same position, we have simulated a tungsten leaf MLC with a leaf height of 10 cm and a source distance of 45 cm. This would have a transmission of the primary radiation beam of only 0.002 %. This tungsten collimator did not have any tongues and grooves in the MC modeling but had rounded leaves. Since it was at the same position of the tray holder it would have the fluence modifications and penumbral changes and even leakage through the closed leaves. To study the leakage characteristics, in this simulation, MLC leaves were adjusted to a 4 cm × 4 cm opening while the jaws were fixed at 10 cm × 10 cm size. While the simulation of an irregularly shaped field would be more realistic, proper analysis cannot be made without the availability of accurate measured data and hence the choice of a regular small field inside the 10 cm × 10 cm field. In addition, arbitrary shaped irregular fields cannot have predictable penumbra because of complex scatter contributions.

## Results

**Figure 5:**
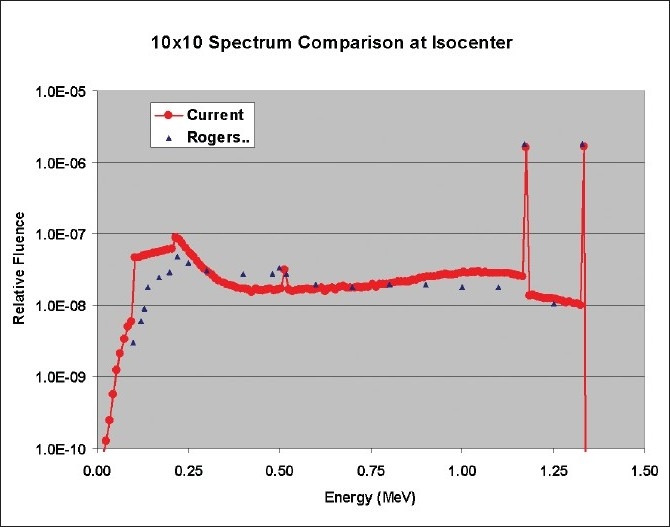
Comparison of 10 cm × 10 cm beam spectrum at isocenter with data from Mora, et al.[4]

**Figure 6:**
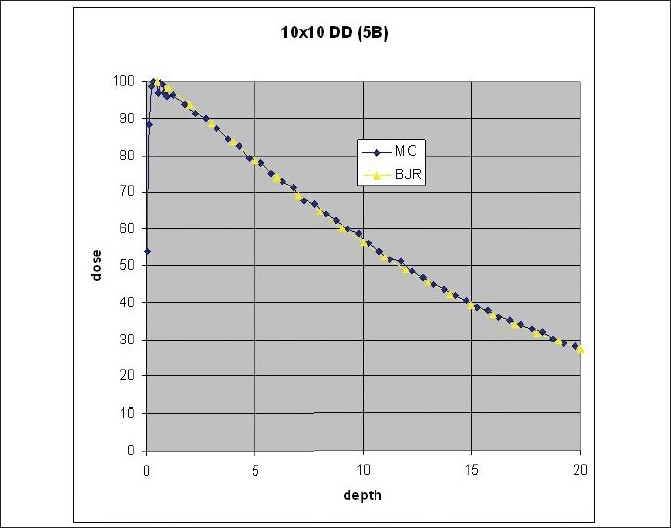
Comparison of 10 cm × 10 cm field depth dose calculation with BJR 25 data; MC calculations were done for five billion histories

**Figure 7:**
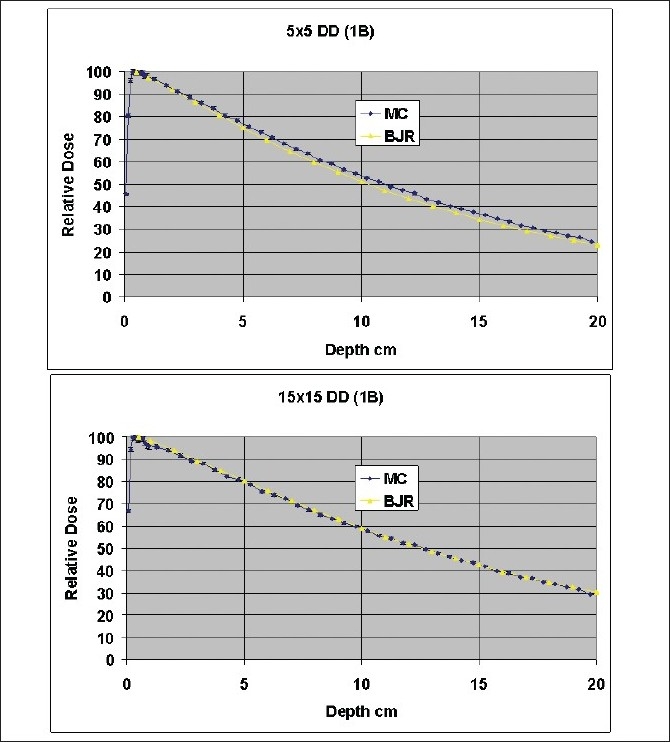
Comparison of 5 cm × 5 cm and 15 cm × 15 cm field depth dose calculations, using 1 billion histories, with BJR 25 data

Figure 5 shows a comparison of 10 cm × 10 cm beam spectrum at isocenter with data from Mora *et al*.[4] It can be seen that there is close agreement between the two spectra. Deviations could be due to differences between simulation dimensions of the source and housing. Figure 6 compares 10 cm × 10 cm field depth dose calculation results of direct BEAMnrc simulation with 5 billion histories compared with standard depth dose tables for cobalt beams from BJR25.[11] The averaged agreement was within 1%. Figure 7 shows a comparison of 5 cm × 5 cm and 15 cm × 15 cm field depth dose calculations using 1 billion histories with BJR25[11] data. The agreement for 15 cm × 15 cm data was within 1%. However, the deviation with 5 cm × 5 cm data was nearly 2%.

**Figure 8:**
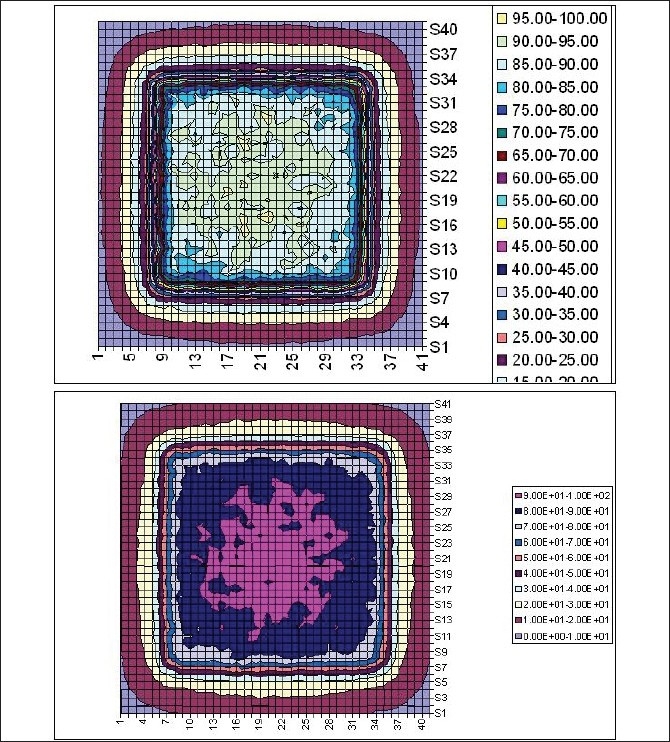
Isodose distributions for a 10 cm × 10 cm field at 4.75 cm and 14.75 cm depths

**Figure 9:**
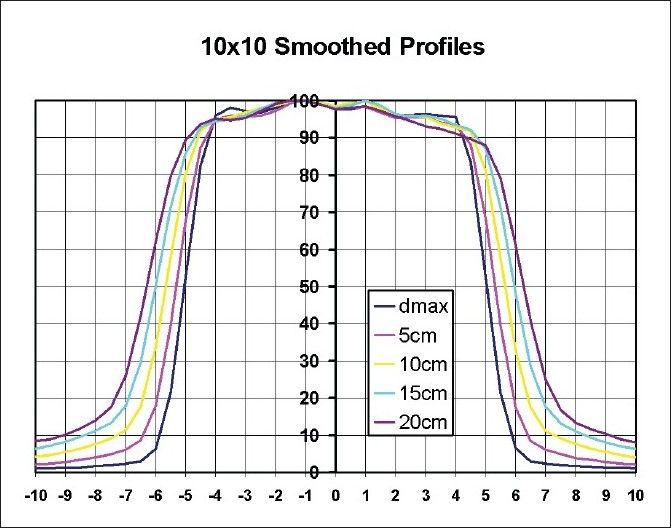
Beam profiles for 10 cm × 10 cm field at depths of dmax, 5 cm, 10 cm 15 cm and 20 cm depths

Figure 8 shows isodose distributions for a 10 cm × 10 cm field at 4.75 cm and 14.75 cm depths. In comparison with shallow depth, there was a blooming effect of the isodose lines at larger depth as expected. Figure 9 shows beam profiles for 10 cm × 10 cm field at depths of d_max_, 5 cm, 10 cm, 15 cm and 20 cm depths. Since the voxel size was relatively large, there was noise in the data due to lack of resolution and lack of adequate statistics. Hence, smoothed profile data has been presented.

**Figure 10:**
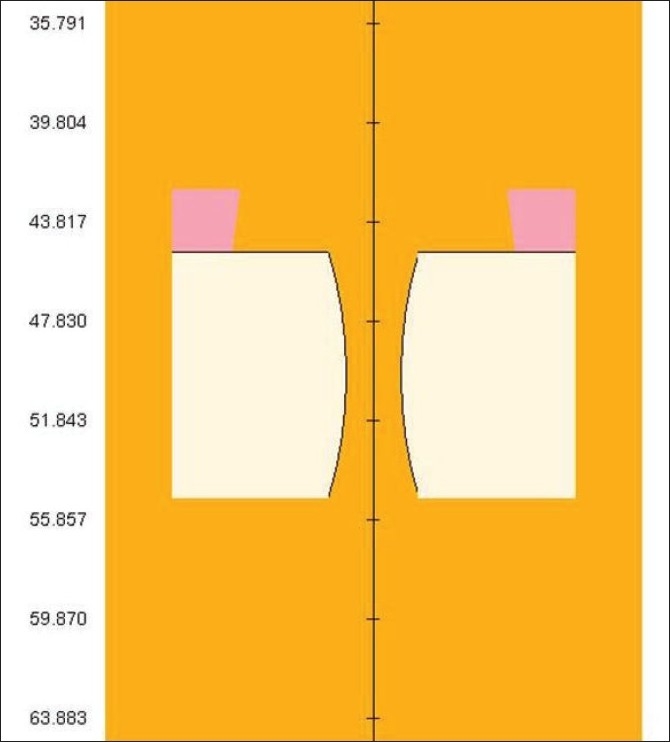
Typical leaf position for 4 cm × 4 cm opening, tungsten leaf of height 10 cm (Secondary jaws were set at 10 cm × 10 cm)

Figure 10 shows typical leaf position for a 4 cm × 4 cm opening for the tungsten leaves of 10 cm height and when the inner and outer jaws were set to 10 cm × 10 cm field.

**Figure 11:**
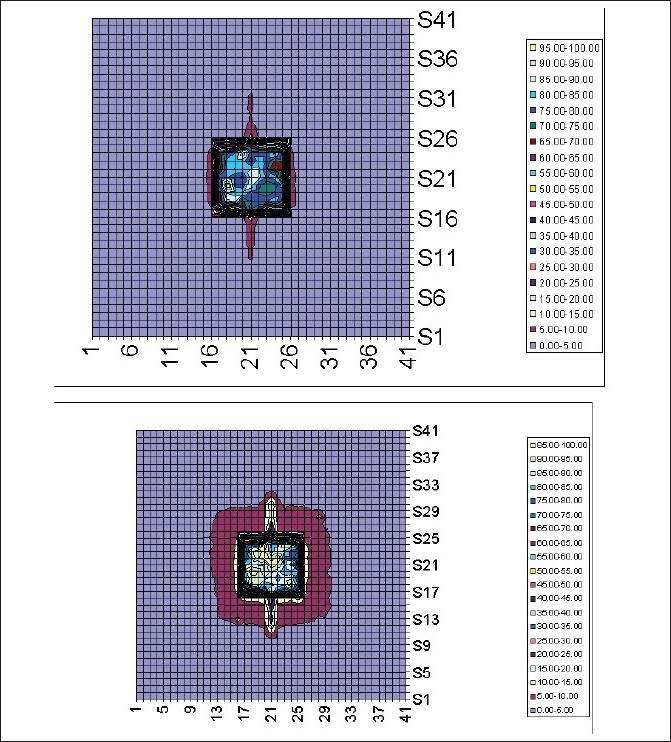
Comparison of isodose distributions for tungsten leaf MLC and LMPA leaf MLC

Figure 11 shows the comparison of isodose distributions for the tungsten leaf MLC and the LMPA leaf MLC. It can be seen that the LMPA distribution has a wider 5% isodose surface compared to the tungsten plot. Roughly, the 5% isodose has spread around the 4 × 4 field size by nearly 1.5 cm all around. There was not much difference in the isodoses in the central 4 × 4 area. More accurate observations can be done by plotting the profiles as described below.

**Figure 12:**
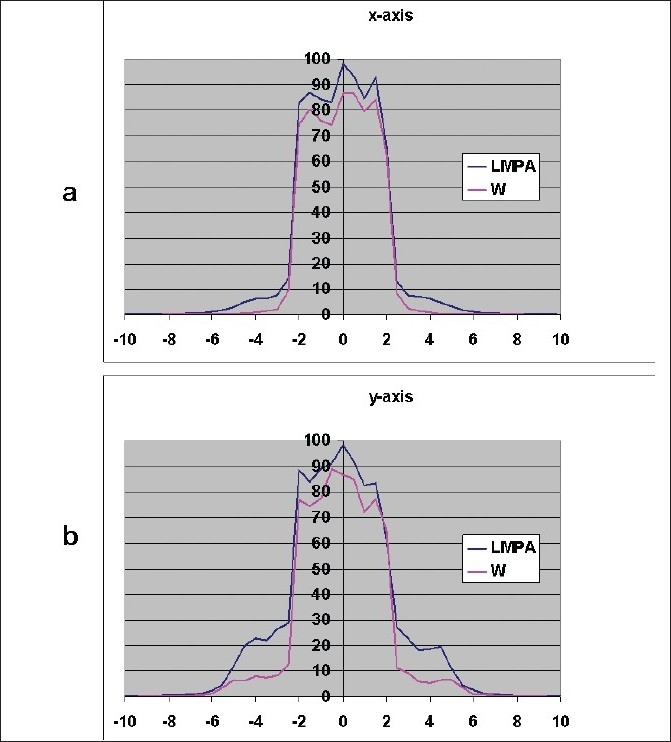
Beam profile comparison of tungsten leaf MLC with LMPA leaf MLC at dmax for a 10 cm × 10 cm field with 4 cm × 4 cm opening in the MLC. a) x- axis profile b) y-axis profile.

Figure 12 shows the radiation characteristics, plotted as profiles, of the proposed LMPA leaves compared with tungsten leaves. From the x-axis profile it was observed additional radiation leakage due to the LMPA collimator was about 4% outside the defined field. As shown in the profiles of the y-axis, the leakage of LMPA compared with tungsten adds an additional 10% leakage in the area of leaf end transmission.

## Discussion

As shown in Figures 6–7, the percent depth dose data obtained from the current simulation closely matched the data from BJR25. The BJR25[11] data for depth dose are considered a good average standard for these machines. Differences if any from the BJR25 data can be attributed to differences in individual machines. A comparison of differences in scatter factors was recently presented by Senthilkumar and Ramakrishnan[12] and previously by Sharma *et al*.[13] According to these studies, although scatter factors differ by up to 2% due to electron contamination, the depth dose data are supposed to be very similar if not identical. The deviation of 2% from BJR25[11] data we have observed with 5 cm × 5 cm data needs further investigation especially for small field sizes. This could be important especially for irregular MLC fields.

Most Linear accelerators are now fitted with MLC. The advantages of MLC in limiting the radiation field to the tumor shape are well known. A recent article by Adams and Warrington[14] compared several conformal and IMRT plans between cobalt and linear accelerator. They have demonstrated that high quality radiotherapy treatments for cobalt units would be feasible if the cobalt unit were to be fitted with proper beam blocking and compensating systems.

In addition, the use of MLC in principle, allows the cobalt unit to be used for intensity modulated radiation therapy (IMRT). There was only one commercial MLC known as WIMRT (WIMRT DMLC by TOPSLANE International, Clearwater, Florida, USA. Distributed by Acceletronics, Mumbai, India) from Germany, designed for linacs, that was advertised as suitable for cobalt machines. This was designed for radiosurgery and IMRT applications with 3 mm leaf width and can cover only 12 cm of width.

There are no known publications on any MLC for cobalt machines except on an indigenous development from India.[15] The paper demonstrated that cobalt conformal plans with MLC can match similar plans from linac. This MLC produced a limited projected field size of only 13 cm × 13 cm at the isocenter.

Although there are many ways to design MLC, the current design aims to retrofit the collimator to any cobalt machine quickly without requiring alteration of the machine. It entails the MLC mounted on the tray holder. This puts a limitation on the design and hence compromises the properties of the MLC. Also the collimator would be bulky compared to a collimator built into the machine at the time of machine fabrication rather than retrofit.

Most MLC designs use tungsten leaves. However, tungsten is expensive and hard to machine. Since most radio surgery cones are made using LMPA, this material was chosen for the prototype design of the leaves. Our experience with LMPA has been that very smooth surface and tongue and groove construction was possible by carefully pouring the blocks and further machining on a milling machine. The material can be sprayed with a thin plastic coat for additional durability. When lead is used for the 6 cm thick MLC leaves, only 2% of the primary radiation of cobalt-60 with an average energy of 1.25 MeV would be transmitted; whereas with LMPA, a 3.5% transmission would result. Although lead offers this advantage, the metal is relatively soft. A steel foil could be used as a covering for lead leaves. Although steel foil covering will slightly increase the transmission penumbra, its incorporation may not adversely affect the overall radiation characteristics of the beam. However, making a tight steel covering can be an engineering task.

It was encouraging to find in this study that there was no visible increase in penumbra of the beam when the beam profiles shapes of tungsten and LMPA were compared as can be seen from Figures 11–12. However, LMPA showed a leakage of nearly 4% assessed from the blocked area.

When two opposite rounded leaves join together, the junction would have less material to attenuate the primary beam. This created a large amount of leakage but was very narrow in extent. This leaf end transmission is usually only about 2% for linear accelerators due to the very small source size. For the current simulation with the cobalt teletherapy machine, this effect is significant. This was as expected and can be eliminated by using the primary collimator to block the leaf gap form opposite leaves. Since cobalt machines do not have asymmetric jaws, the leaf-end transmission can only be partially reduced.

In conclusion, it was possible to generate accurate data for treatment planning purposes using the MC approach. The MC simulation has proved helpful to evaluate MLC design for cobalt-60 teletherapy machine. Without the use of such simulation it was not possible to assess the radiation properties of the MLC design especially near beam edges where critical structures could lie.

While this study focused on finding only leakage properties, further studies are needed to determine photon fluence modifications made by the MLC for various field configurations. After completion of the proposed MLC fabrication, another MC simulation would be desirable, using the exact fabricated parameters, to compare the simulation with measurements.

## APPENDIX

In this appendix, the BEAMnrc specific parameter files are described.

BEAMnrc comes with a graphic user interface (GUI). The following figure is the first screen of the GUI.

This GUI allows one to build the accelerator using building blocks like target, flattening filter, ion chamber, mirror, jaws etc. This is done through the menu item **File**.

After building the components one can **Preview** the accelerator which draws each component to relative scale and displays the total picture. Then, the software is assembled and compiled internally. The next phase is to run the accelerator through **Execute** command; the beam transport is carried till the specified number of histories. After the execution, the user analyzes the output files. The output file may contain the dose calculations in the central axis of a phantom. In addition, the problem can be defined to produce a phase space file at any plane of the accelerator. The phase space files can be further processed by another set of modules.

The following figure shows the expansion of the menu item **File**.

Here, one can specify a new accelerator and save it or load a saved accelerator file. Specifying the accelerator involves only specifying the building blocks or component modules. After the accelerator is built, one needs to define the physical dimensions of the components and this is done through an input file. The PEGS4 software menu allows one to define component materials made of elements, compounds and mixtures. The PEGS4 collects the corresponding attenuation data at various photon energies.

For example, to build a cobalt machine, the cobalt source can be modeled as a cylinder. In the component module one can select circular SLABS to define cylinders. In the input file the dimensions of the cylinder are defined. The material COBALT can be selected in PEGS4.

In the following, the step I referred to in the paper is configured. There are four component modules; SLABS for the housing, CONESTAK for the source, SLABS for air gap and PYRAMIDS for the primary collimator.

The following screen shows how the input parameters are defined.

For the cobalt source, item 3 as shown below is selected. Each parameter entry has description associated with it, which can be seen by clicking the question mark

The following screen defines the source as a cylinder with radius of 0.75 cm and height of 2.0 cm. The source photons are sampled from a bare cobalt-60 spectrum given as a file.

The four modules built above are previewed as below

**THE INPUT FILE**

The following is the actual input file. The reader is referred to the documentation of the software for proper definition and description of parameters. Briefly, on line 4, the number of histories was defined as 1.7 billion. The starting random number seeds are 33, 97. Thousand hours are given for the maximum computation hours allowed for this problem. The next line has all the source parameters in terms of size. Line 6 defines a spectrum and the next line for the location of the spectrum file. Line 9 has EGS transport parameters which are default values. Line 11 defines a scoring plane after component 4 i.e. the end of the primary collimator. Scoring implies a phase space file will be created. The start of the coordinate system is the bottom of the source cylinder. All Z values above this plane are negative. −5.67cm is the start of the problem geometry. The source is between −0.1 and −2.15 cm with a radius of 0.75 cm.

**Table:**

Cobalt-60	#!GUI1.0
AIR700ICRU	
0, 0, 1, 0, 0, 2, 0, IWATCH ETC.	
1700000000, 33, 97, 1000, 0, 0, 0, 0, NCASE ETC.	
0, 3, 0, 0.75, -2.15, -0.1, 0.0, 0.0, 0.0, 0.0, IQIN, ISOURCE + OPTIONS	
1, SPECTRUM	
C:/HEN_HOUSE/spectra/bareco60.spectrum	
1	
0, 0, 0.7, 0.01, 0, 0, , 0 , ECUT, PCUT, IREJCT, ESAVE	
0, 0, 0, 0, 0, PHOTON FORCING	
1, 4, SCORING INPUT	
5, 0	
1, 2, 3, 4, 5,	
0, DOSE COMPONENTS	
-5.67, Z TO FRONT FACE	

The following are the parameters of the first module which is a slab of lead	
The radius of the slab is defined as 10 cm and the thickness is 3 cm.	
The material as mentioned above, is lead. It starts at a z value of −5.67cm to −2.67 cm	

*********** start of CM SLABS with identifier housing ***********	
10, RMAX	
lead back of source	
1, NSLABS	
-5.67, ZMIN	
3, 0.7, 0.01, 0, 0, 0	
PB700ICRU	

The following is the second module identifying the cobalt source capsule. The source is defined as a cylinder of radius 0.75 cm and height 2.05 cm. Three layers of thickness 0.52 cm, 2.05 cm and 0.1 cm are defined. Each layer has a central cylinder and 2 concentric hollow cylinders. Together they make a solid structure. The inner cylinder has a radius of 0.75 cm, the next cylinder extends this to 1.0 cm and the outer cylinder has on outer radius of 10 cm. The top layer is made up of steel, steel and lead. The middle layer is made up of cobalt, steel and lead. The bottom layer has steel, steel and lead.

See the figure below.

**Table:**

*********** start of CM CONESTAK with identifier source ***********
10, RMAX
iron cladding and lead housing
-2.67, 1.0, ZMIN, RBN
3, NUMBER OF LAYERS
0.52, 0.75, 0.75,
2.05, 0.75, 0.75,
0.1, 0.75, 0.75,
0.7, 0.01, 0, 0, OUTER WALL
PB700ICRU
0.7, 0.01, 0, 0,
FE700ICRU
0.7, 0.01, 0, 0,
FE700ICRU
0.7, 0.01, 0, 0,
Cobalt
0.7, 0.01, 0, 0,
FE700ICRU
0.7, 0.01, 0, 0,
FE700ICRU
0.7, 0.01, 0, 0,
FE700ICRU

The third module is a layer of air defined as a slab of thickness 1.5 cm. It starts at the bottom of the source capsule at a Z distance of 0.0 cm.

*********** start of CM SLABS with identifier air ***********

10, RMAX

: Start MC Transport Parameter:

1, NSLABS

0, ZMIN

1.5, 0.7, 0.01, 0, 0, 0

AIR700ICRU

The following are the parameters for the primary collimator which is a rectangular shaped pyramid. Line 5 describes the collimator z value starts at 1.5 cm and ends at 7.7 cm. The top of the collimator has a square opening of 3.4 cm × 3.4 cm and the bottom of the collimator opening is 5.9 cm × 5.9 cm. Line 8 specified the collimator is made of tungsten. The outer dimension of collimator is 10 cm.

**Table:**

*********** start of CM PYRAMIDS with identifier primary collimator ***********
10, RMAX
primary jaw
1, 0, #LAYERS, AIR OUTSIDE
1.5, 7.7, 1.7, 2.95, -1.7, -2.95, 1.7, 2.95, -1.7, -2.95, 20, 20,
0.7, 0.01, 0, 0, ECUT ETC. FOR AIR
0.7, 0.01, 0, 0,
W700ICRU
*********************end of all CMs*****************************

The following parameters are general EGS parameters

#########################

: Start MC Transport Parameter:

Global ECUT= 0.7

Global PCUT= 0.01

Global SMAX= 5

ESTEPE= 0.25

XIMAX= 0.5

Boundary crossing algorithm= PRESTA-I

Skin depth for BCA= 0

Electron-step algorithm= PRESTA-II

Spin effects= On

Brems angular sampling= Simple

Brems cross sections= BH

Bound Compton scattering= Off

Pair angular sampling= Simple

Photoelectron angular sampling= Off

Rayleigh scattering= Off

Atomic relaxations= Off

Electron impact ionization= Off

:Stop MC Transport Parameter:

#########################
